# A Rare Case of Synovial Sarcoma of the Prostate Causing Urinary Retention

**DOI:** 10.7759/cureus.21057

**Published:** 2022-01-09

**Authors:** Syed Ehsanullah, Syeda Zarmeena Rashid, Adeel Haq, Saiyed Abdullah A Ehsanullah

**Affiliations:** 1 Medicine, Washington University School of Medicine, Saint Louis, USA; 2 Internal Medicine, Dow University of Health Sciences, Dow International Medical College, Karachi, PAK; 3 Radiology, Washington University School of Medicine, Saint Louis, USA; 4 Internal Medicine, Ziauddin University, Karachi, PAK

**Keywords:** prostate carcinoma, urinary obstruction, sarcoma, synovial sarcoma of the prostate, synovial sarcoma

## Abstract

Synovial sarcomas of the prostate are exceedingly rare malignant tumors. Only a few cases have been reported so far. We discuss a case of a 52-year-old male who presented with signs and symptoms of benign prostate hyperplasia (BPH) and was diagnosed with synovial sarcoma of the prostate. Since this sarcoma is rare, it can easily be misdiagnosed with BPH or adenocarcinoma of the prostate.

## Introduction

Leiomyosarcoma is the most common type of soft tissue sarcoma. Soft tissue sarcoma is a soft tissue carcinoma primarily seen in young adults in the para-articular region of the extremities. It can also present in unusual sites like the orofacial or oropharyngeal region, larynx, esophagus, lung, heart, abdominal wall, gastrointestinal tract, and retroperitoneum [1-2]. Several sarcomas have previously been described in the prostate, including fibrosarcoma, rhabdomyosarcoma, leiomyosarcoma, and stromal sarcoma. However, synovial sarcoma in the prostate is extremely rare. Only seven previously reported cases have been published [3-8].

## Case presentation

**Figure 1:**
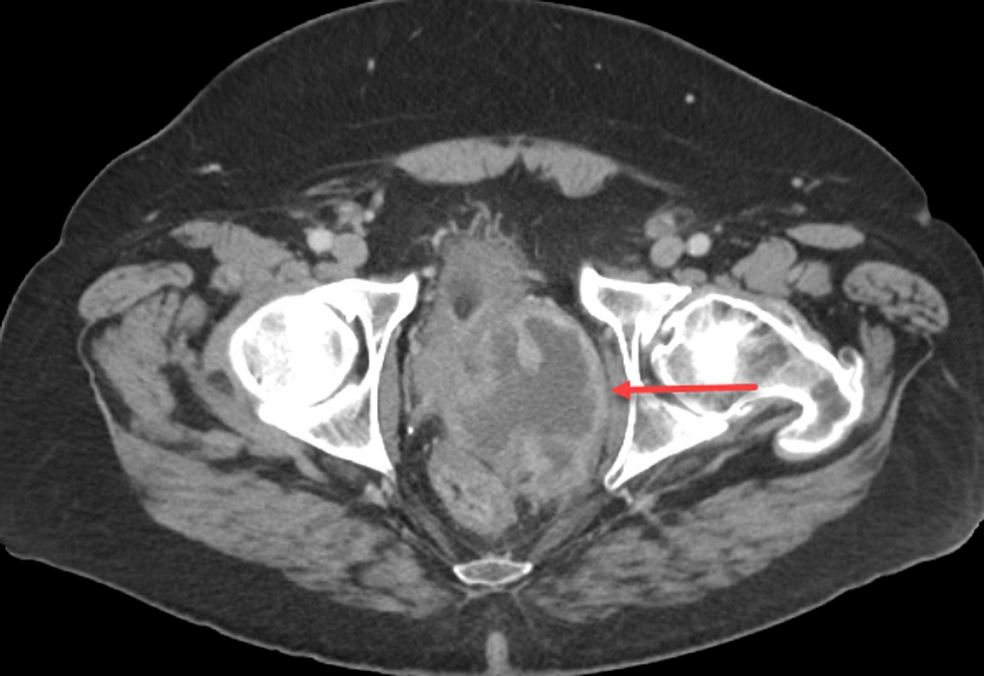
Axial CT pelvis showing enlarged prostate (9.6cm) (red arrow) with a mass effect on the rectum and bladder

**Figure 2:**
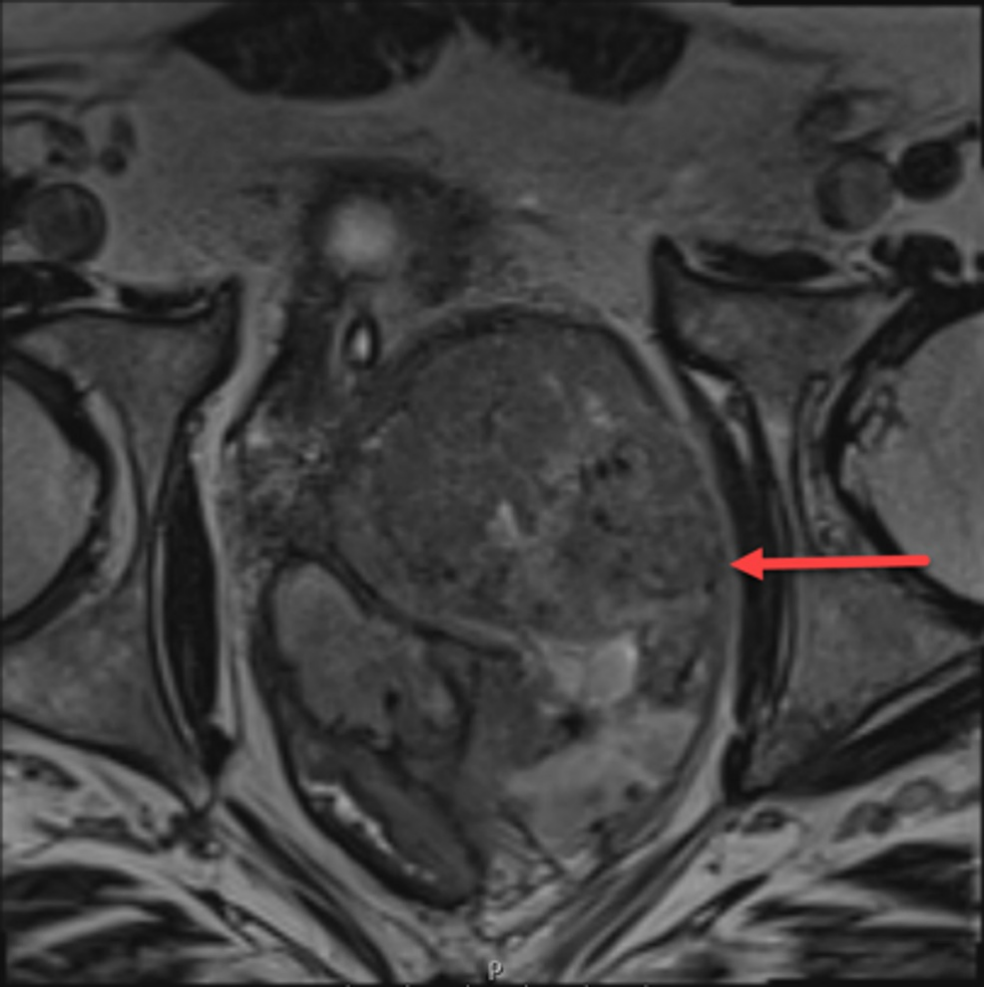
Axial MRI T2 showing heterogeneous necrotic mass arising from the left prostate (red arrow), invading the rectal wall

**Figure 3:**
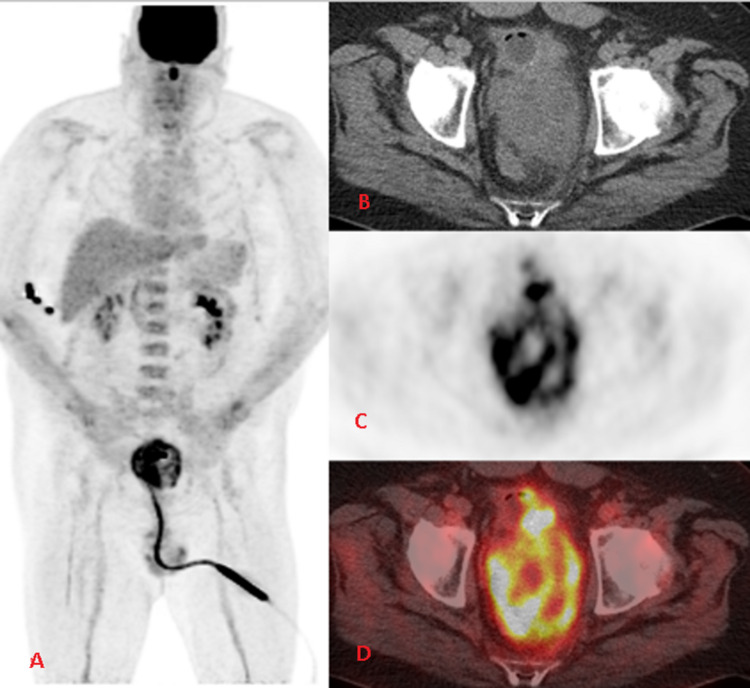
(A) FDG PET MIP showing heterogeneous FDG uptake in the prostate gland in addition to the physiologic distribution of the radiotracer; (B) Axial non-contrast CT showing heterogeneously enlarged prostate gland with interspersed areas of necrosis; (C) Axial FDG PET showing heterogeneous FDG uptake in the enlarged prostate gland; (D) Fused FDG PET CT showing heterogenous marked tracer uptake in the enlarged irregular prostate gland FDG: fluorodeoxyglucose; PET: positron emission tomography; MIP: maximum intensity projection

A 52-year-old male patient with past medical history significant for nephrolithiasis and gout, who initially presented to the emergency department with the complaint of progressively worsening urinary retention, started as difficulty initiating urination and later developed complete urinary retention over several months. In the ED, a CT abdomen/pelvis contrast scan was performed, demonstrating enlarged prostate (9.6cm) with a mass effect on the rectum and bladder (Figure 1). A foley catheter was inserted, and the patient was discharged from ED to follow up with urology as an outpatient. A transrectal ultrasound (TRUS)-guided biopsy was performed, which demonstrated spindle cell malignancy with immunostaining positive for *TLE1* gene and negative for CD117, CD34, progesterone receptors (PR), desmin, SOX10, STAT6, pan-cytokeratin, and AE1/AE3. An MRI of the pelvis showed a large heterogeneous partially necrotic mass arising from the left prostate, invading the rectal wall, suspicious for prostatic sarcoma (Figure 2). A positron emission tomography (PET) showed 7.2 x 7.6 cm heterogeneously markedly fluorodeoxyglucose (FDG)-avid necrotic mass with areas of hemorrhage encompassing the entire prostate. Mildly avid FDG uptake in left external iliac lymph node and left common iliac lymph node was noted on PET scan, concerning the local nodal metastatic disease (Figure 3).

Due to the case's complexity, pathology slides were sent for a second opinion. The final immunostaining detected the *SS18-SSX2* fusion gene consistent with a diagnosis of synovial sarcoma of the prostate. The patient was started on chemotherapy on Adriamycin®, ifosfamide, and mesna (AIM) therapy with a plan to repeat every 21 days; a total of six complete cycles.

## Discussion

Synovial sarcoma is exceptionally uncommon and accounts for 5-10% of soft-tissue tumors. For every one million people, only one to two are diagnosed with synovial sarcoma per year in the United States [9]. About 90% of synovial sarcomas occur on the extremities. In the genitourinary system, most synovial sarcomas have been reported in the kidney [10].

Synovial sarcoma typically presents in young to middle-aged men. Most patient presents with increased urinary frequency, hematuria, dysuria, and nocturia before urinary retention due to bladder outlet obstruction. CT scan and MRI imaging modalities help stage tumor extent and plan surgical resection. MRI is the modality of choice for the initial staging of synovial sarcoma because of the information provided by intrinsic signal characteristics and superior soft-tissue contrast. MRI is also required to assess the extent and inherent characteristics of the sarcoma. Diagnosis of synovial sarcoma of the prostate is mainly dependent on histopathology and immunostaining of the mass. The *SS18-SSX1 *and *SS18-SSX2* fusion genes are exclusive in synovial sarcomas. Detection of these fusion genes confirms the diagnosis of synovial sarcoma [11]. As in our case, prostate biopsy demonstrated spindle cell malignancy with immunostaining positive for *SS18-SSX2* fusion gene, confirming the diagnosis of synovial carcinoma of the prostate.

Synovial sarcoma is a malignant and aggressive tumor with a poor prognosis [7]. It spreads either by systemic spread or through local invasion. The mean survival is of 24 months despite multimodal treatment [12]. Prostate-specific antigen (PSA) is periodically monitored to see the treatment response in prostate adenocarcinoma but has no role in prostate sarcomas. PSA levels are usually within normal limits in synovial sarcoma of the prostate due to their non-epithelial origin [13]. Treatment of prostate sarcoma is uncertain due to limited reported cases and experience, but it appears that aggressive resection must be considered for the treatment of locally confined synovial sarcoma [6]. Other strategy includes surgical resection with adjuvant chemotherapy and/or radiotherapy if external iliac, hypogastric, or distant lymph nodes are involved because of its aggressive nature and poor prognosis [3,14].

## Conclusions

The prostate is a rare and unusual location for the synovial sarcoma. Since the incidence of this sarcoma is very rare, it can easily be misdiagnosed with BPH and adenocarcinoma of the prostate because of similar signs and symptoms. We present this case to elevate the awareness about synovial sarcoma of the prostate; a physician should always keep it as a differential diagnosis for prostate enlargement in young and middle-aged men.
